# Association of changes in frailty status with the risk of all-cause mortality and cardiovascular death in older people: results from the Chinese Longitudinal Healthy Longevity Survey (CLHLS)

**DOI:** 10.1186/s12877-024-04682-2

**Published:** 2024-01-25

**Authors:** Ziqiong Wang, Haiyan Ruan, Liying Li, Ningying Song, Sen He

**Affiliations:** 1 Department of Cardiology, West China Hospital of Sichuan University, No.37 Guoxue Alley, Chengdu, China; 2 Department of Cardiology, Hospital of Traditional Chinese Medicine, Shuangliu District, Chengdu, China; 3 Department of Otolaryngology-Head & Neck Surgery, West China Hospital, Sichuan University, No.37 Guoxue Alley, Chengdu, China

**Keywords:** All-cause mortality, Cardiovascular death, Frailty transitions, Older people

## Abstract

**Background:**

Few studies have investigated the association between changes in frailty status and all-cause mortality, inconsistent results were reported. What’s more, studies that evaluated the effect of changes of frailty on cardiovascular death in older population are scanty. Therefore, the present study aims to investigate the association of such changes with the risk of all-cause mortality and cardiovascular death in older people, using data from the Chinese Longitudinal Healthy Longevity Survey (CLHLS).

**Methods:**

A total of 2805 older participants from two consecutive waves (i.e. 2011 and 2014) of the CLHLS were included for analysis. Based on the changes in frailty status from wave 2011 to wave 2014, participants were categorized into 4 subgroups, including sustained pre/frailty, robustness to pre/frailty, pre/frailty to robustness and sustained robustness. Study outcomes were all-cause mortality and cardiovascular death, and Cox regression analysis examined the association of changes in frailty status with outcomes.

**Results:**

From wave 2011 to wave 2014, 33.2% of the participants had frailty transitions. From wave 2014 to wave 2018, there were 952 all-cause mortalities and 170 cardiovascular deaths during a follow-up of 9530.1 person-years, and Kaplan-Meier analysis demonstrated that cumulative incidences of the two outcomes were significantly lower in more robust participants (all log-rank p < 0.001). Compared with the subgroup of sustained pre/frailty, the fully adjusted HRs of all-cause mortality were 0.61 (95% CI: 0.51–0.73, p < 0.001), 0.51 (95% CI: 0.42–0.63, p < 0.001) and 0.41 (0.34–0.49, p < 0.001) in the subgroup of robustness to pre/frailty, the subgroup of pre/frailty to robustness, and the subgroup of sustained robustness, respectively. The fully adjusted HRs of cardiovascular death were 0.79 (95% CI: 0.52–1.19, *p* = 0.256) in the subgroup of robustness to pre/frailty, 0.45 (95% CI: 0.26–0.76, *p* = 0.003) in the subgroup of pre/frailty to robustness and 0.51 (0.33–0.78, *p* = 0.002) in the subgroup of sustained robustness when comparing to the subgroup of sustained pre/frailty, respectively. Stratified analysis and extensive sensitivity analyses revealed similar results.

**Conclusions:**

Frailty is a dynamic process, and improved frailty and remaining robust are significantly associated with lower risk of all-cause mortality and cardiovascular death in older people.

**Supplementary Information:**

The online version contains supplementary material available at 10.1186/s12877-024-04682-2.

## Introduction

Frailty is an age-related biological syndrome, which is characterized by a decline of physiological function and an increase of vulnerability to endogenous and exogenous stressors [1]. Previous studies indicated that the prevalence of frailty increased with age and ranged from 4% to 59% in different community-dwelling elderly populations [2]. With the rapid ageing population worldwide [3], the prevalence of frailty is predicted to raise further [4]. Therefore, the issue of frailty has received considerable attention among older people due to mounting evidence showed that it could lead to serious consequences for their physical and psychological health [5], finally increasing the risk of mortality [6, 7].

Although ageing is strongly associated with frailty, but frailty is a dynamic process involving both improvement and progression, but not an irreversible one-way process to disability or death. A previous study revealed that among 42,775 community-dwelling older people with a mean follow-up of 3.9 years, there were 13.7% had improved frailty status, 29.1% had worsened frailty status and 56.5% maintained the same frailty status [8]. One can expect that the prognostic value of different frailty transitions for health outcomes, such as mortality should be much better that that of a single assessment of frailty status in older people. However, important gaps remain. First, to our knowledge, there were only few studies that examined the association between changes in frailty status and mortality in older people [9–12]. Second, the results of these studies were inconsistent [9–12], and the sample size for some studies was less than 1000 [9, 10]. Third, the outcome mainly focused on all-cause mortality [9–12], not including other important outcomes, such as cardiovascular death. As we know, the value of frailty as a prognostic marker has been well demonstrated in a broad spectrum of cardiovascular diseases [13]. However, studies that evaluated the effect of changes of frailty on cardiovascular death in older population are scanty. Therefore, evidence between frailty transitions and health outcomes, especially cause-specific death needs to be enhanced in older people.

Based on the above reasons, this study aims to determine the association of changes in frailty status with the risk of all-cause mortality and cardiovascular death in older people using data from the Chinese Longitudinal Healthy Longevity Survey (CLHLS).

## Methods

### Study participants

**Fig. 1:**
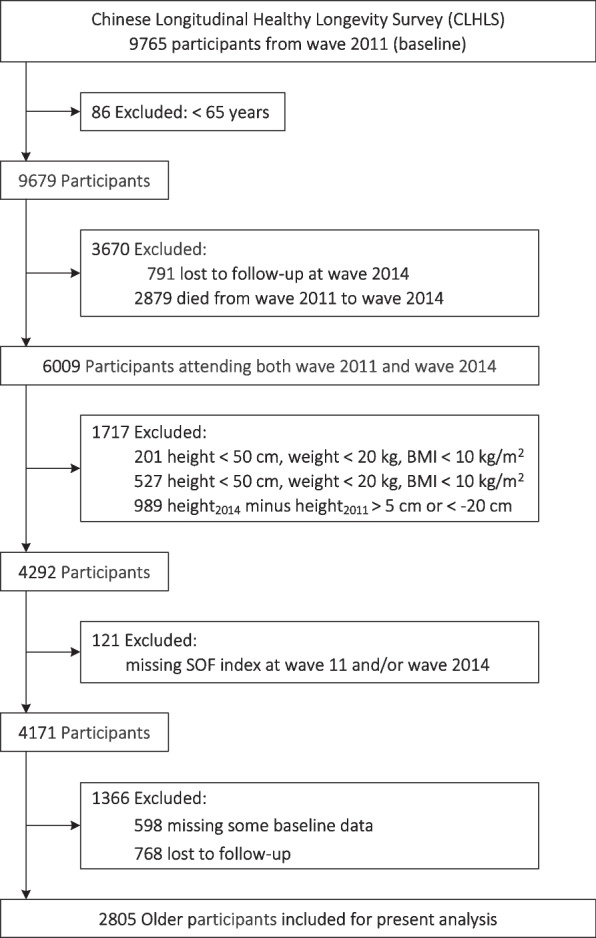
Flow chart. Potential abnormal values were excluded, such as height < 50 cm, weight < 20 kg, or BMI < 10 kg/m^2^. Generally, height should be smaller with age; therefore, if height at wave 2014 minus height at wave 2011 was significantly abnormal, such as < -20 cm or > 5 cm, it was also excluded. Abbreviations: BMI = body mass index; SOF = Study of Osteoporotic Fractures

The CLHLS is a nationwide, ongoing, prospective cohort study of community-dwelling Chinese older people, and aims to better understand the determinants of healthy longevity among the oldest Chinese aged 80 and above, and people as well as that of younger old people aged 65–79. It began in 1998, with subsequent follow-up in 2000, 2002, 2005, 2008, 2011, 2014, and 2018. To reduce the attrition due to death and loss to follow-up, new participants are enrolled during the following waves from 1998. The CLHLS is conducted in a randomly selected half of the counties and cities in 23 of the 31 provinces, covering about 85.0% of Chinese population, and administered in participants’ homes by trained interviewers. More details about CLHLS have been reported elsewhere, and data quality was reported to be generally good [14]. Because the frailty status used in the present study was collected since wave 2011, participants who completed the evaluations of frailty status for two consecutive waves (i.e. 2011 and 2014) were included in the present study. Then, participants were followed up until wave 2018. Figure 1 depicts the recruitment process, and the final sample consisted of 2805 older people (age ≥ 65 years).

The CLHLS was carried out in accordance with the principles of the Declaration of Helsinki, and was authorized by the research ethics committee of Peking University (IRB00001052-13074). Before participating, survey respondents provided their informed consent.

### Assessment of frailty status

Frailty status was defined by the study of osteoporotic fractures (SOF) index [15, 16]. Based on the data available in the CLHLS, a modified SOF index [17]was used in the present study, and it included 3 components: (1) underweight (body mass index < 18.5 kg/m^2^); (2) muscle strength (inability to stand up from a chair without the assistance of arms); and (3) low energy level (indicated by a positive response to the question "Over the last 6 months, have you been limited in activities because of a health problem?"). Then, frailty status was categorized into three states: frailty (2 or 3 components), prefrailty (1 component), and robustness (0 component).

### Assessment of other covariates

eTable 1 shows the detailed information of other covariates, which were obtained through questionnaires, including: age, sex, education, marital status, income, residence, living with family, current smoking, current drinking, current exercise, regular intake of foods (fruits, vegetables, meats, fishes, eggs, and beans), comorbidities (hypertension, diabetes, heart diseases, cerebrovascular diseases, respiratory diseases, and cancer), and activities of daily living (ADL) disability. More detailed information about these covariates can be found on: https://agingcenter.duke.edu/CLHLS.

### Study outcome

The study outcome was all-cause mortality and cardiovascular death, and the International Classification of Diseases (ICD, 10th revision) was used to assess participants` underlying cause of cardiovascular death (codes I00-I99). The date and cause of death of a deceased participant were collected from the participant’s closest relatives and were certified from the village doctor though death certificates, hospital admission records, medical records if available, especially for the cause of death. In the CLHLS, the reliability of mortality may be more reliable than those obtained from the national census, although some recall errors occurred [18].

### Statistical analysis

The missing values for all the baseline variables were no more than 4.51%, and eTable 2 shows the distributions of variables with missing data. Due to relatively low missing rates, we deleted the cases with missing data in the main analyses, with multiple imputation as a sensitivity analysis. Overall, we conducted the analyses with the following steps: (1) comparisons of baseline data; (2) evaluating adjusted risk of changes in frailty status for cardiovascular death and all-cause mortality; (3) performing stratified and sensitivity analyses to examine the robustness of main findings; (4) conducting additional analyses to expand the main results.

**Fig. 2:**
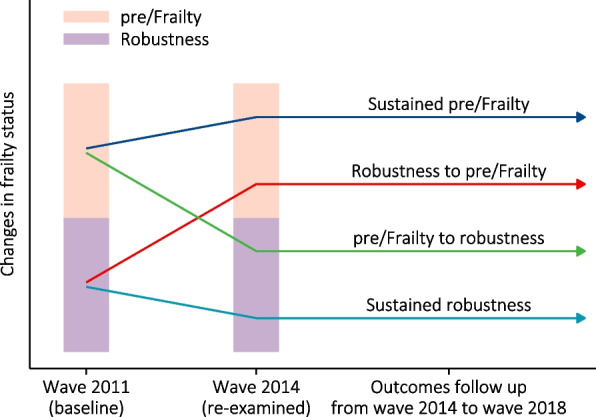
Definitions of changes in frailty status and outcomes follow-up

According to above classifications of frailty status, there should be 9 changes in frailty status from wave 2011 to wave 2014; while, more groups might reduce statistical power based on the sample size (*n* = 2805). Compared to mortality rate in the robustness group (eTables 3 and 4), the mortality rates were higher in the prefrailty and frailty groups, and then we combined the two groups into a new group, namely pre/Frailty, to perform further stratified analysis. Finally, four types of change in frailty status were determined as: (1) sustained pre/Frailty; (2) robustness to pre/Frailty; (3) pre/Frailty to robustness; (4) sustained robustness (Fig. 2). Baseline characteristics were displayed across the four changes in frailty status, and the characteristics were described as median (interquartile range, IQR) for continuous variables and number (percentage) for categorical variables. Kruskal-Wallis test, Chi-square test or Fisher`s exact test was used for comparisons of baseline characteristics as appropriate.

The occurrence of outcomes was estimated using the Kaplan-Meier method, and the log-rank test was used for comparisons. Cox proportional-hazard regression analysis was used to examine the association of changes in frailty status with outcomes, and no evidence of violation of the proportional-hazard assumption was found.

Furthermore, to test the robustness of our findings, we performed a series of stratified and sensitivity analyses, including: (1) stratified analysis assessed the consistency of association between changes in frailty status and outcomes in various subgroups, and interactions were examined by likelihood ratio testing; (2) we examined the prognostic utility of changes in frailty status after controlling for frailty status at wave 2014; (3) to clarify the role of participants lost to follow-up in the associations, we did sensitivity analyses for such participants censored at two time points: median and the end of follow-up; (4) to exclude the deaths that occurred within the first year of follow-up to reduce potential reverse causation; (5) to account for the competing risk between cardiovascular death and all-cause mortality, the Fine-Gray model was fitted to assess the association of changes in frailty status with cardiovascular death; (6) we excluded participants with hypertension or diabetes or heart diseases at baseline to test the robustness of major findings for cardiovascular death, and participants with any comorbidity were excluded to test the robustness of major findings for all-cause mortality; (7) we defined participants with unknown causes of deaths as cardiovascular death to test the robustness of major findings; (8) to mitigate potential bias caused by missing data, we used multiple imputation by chained equations to create 10 datasets, of which the resultant model estimates for each were combined using Rubin’s rules; (9) we also calculated the E-values, which could assess the potential for unmeasured confounding between changes in frailty status and outcomes, and it quantifies the required magnitude of an unmeasured confounder that could negate the observed association between changes in frailty and outcomes.

Finally, to expand the main results, we performed exploratory analyses to examine the associations of changes in two out of three frailty status (i.e. frailty, prefrailty, and robustness) with outcomes, and to assess the associations of improved levels of frailty status with outcomes.

All analyses were performed with R version 4.1.0 including the “compareGroups”, “survival”, “tidyverse”, “rms”, “mice”, “forestplot”, “survminer”, and “stats” packages (http://www.R-project.org). All tests were two sided, and *p* values < 0.05 were considered statistically significant.

## Results

### Baseline characteristics

The sample consisted of 2805 older participants with a median age 80 years (IQR: 73.00, 87.00), and 48.84% were male participants. Baseline characteristics of the participants stratified by frailty transitions are presented in Table 1. Among them, 30% (*n*=832) of the older participants were categorized in subgroup of sustained pre/frailty, 18% (*n*=498) were categorized in subgroup of robustness to pre/frailty, 15% (*n*=432) were categorized in subgroup of pre/frailty to robustness, and 37% (*n*=1043) were categorized in subgroup of sustained robustness. Results revealed significant differences in most of the baseline characteristics, including age, sex, education, marital status, income, residence, current smoking, current drinking, current exercise, regular intake of foods, heart diseases, respiratory diseases, and activities of daily living (ADL) across the four subgroups.

**Table 1:** Baseline characteristics

	All	Sustained pre/Frailty	Robustness to pre/Frailty	pre/Frailty to robustness	Sustained robustness	*p* value
No. of participants	2805	832	498	432	1043	
Sex: male	1370 (48.84%)	306 (36.78%)	240 (48.19%)	204 (47.22%)	620 (59.44%)	<0.001
Age (years)	80.00 (73.00, 87.00)	86.00 (78.00, 94.00)	81.00 (74.00, 88.00)	80.00 (73.00, 87.00)	75.00 (71.00, 81.00)	<0.001
Education						<0.001
No school	1434 (51.12%)	530 (63.70%)	278 (55.82%)	221 (51.16%)	405 (38.83%)	
1 year or more	1371 (48.88%)	302 (36.30%)	220 (44.18%)	211 (48.84%)	638 (61.17%)	
Marital status						<0.001
In marriage	1398 (49.84%)	304 (36.54%)	230 (46.18%)	209 (48.38%)	655 (62.80%)	
Not in marriage	1407 (50.16%)	528 (63.46%)	268 (53.82%)	223 (51.62%)	388 (37.20%)	
Income						0.002
Rich	548 (19.54%)	136 (16.35%)	97 (19.48%)	75 (17.36%)	240 (23.01%)	
Fair/poor	2257 (80.46%)	696 (83.65%)	401 (80.52%)	357 (82.64%)	803 (76.99%)	
Residence						<0.001
Urban	1264 (45.06%)	327 (39.30%)	241 (48.39%)	192 (44.44%)	504 (48.32%)	
Rural	1541 (54.94%)	505 (60.70%)	257 (51.61%)	240 (55.56%)	539 (51.68%)	
Living with family	2273 (81.03%)	671 (80.65%)	391 (78.51%)	358 (82.87%)	853 (81.78%)	0.325
Current smoking	582 (20.75%)	143 (17.19%)	106 (21.29%)	92 (21.30%)	241 (23.11%)	0.018
Current drinking	553 (19.71%)	119 (14.30%)	94 (18.88%)	90 (20.83%)	250 (23.97%)	<0.001
Current exercise	1121 (39.96%)	267 (32.09%)	204 (40.96%)	165 (38.19%)	485 (46.50%)	<0.001
Regular intake of foods						
Fruits	1071 (38.18%)	266 (31.97%)	182 (36.55%)	157 (36.34%)	466 (44.68%)	<0.001
Vegetables	2559 (91.23%)	727 (87.38%)	451 (90.56%)	391 (90.51%)	990 (94.92%)	<0.001
Meats	2076 (74.01%)	602 (72.36%)	374 (75.10%)	301 (69.68%)	799 (76.61%)	0.025
Fish	1236 (44.06%)	346 (41.59%)	216 (43.37%)	174 (40.28%)	500 (47.94%)	0.012
Eggs	1944 (69.30%)	549 (65.99%)	349 (70.08%)	275 (63.66%)	771 (73.92%)	<0.001
Beans	1527 (54.44%)	425 (51.08%)	262 (52.61%)	225 (52.08%)	615 (58.96%)	0.003
Comorbidities						
Hypertension	841 (29.98%)	227 (27.28%)	151 (30.32%)	141 (32.64%)	322 (30.87%)	0.190
Diabetes	114 (4.06%)	32 (3.85%)	22 (4.42%)	9 (2.08%)	51 (4.89%)	0.092
Heart diseases	319 (11.37%)	94 (11.30%)	59 (11.85%)	65 (15.05%)	101 (9.68%)	0.031
Cerebrovascular diseases	179 (6.38%)	59 (7.09%)	29 (5.82%)	30 (6.94%)	61 (5.85%)	0.640
Respiratory diseases	302 (10.77%)	115 (13.82%)	40 (8.03%)	58 (13.43%)	89 (8.53%)	<0.001
Cancer	15 (0.53%)	2 (0.24%)	4 (0.80%)	2 (0.46%)	7 (0.67%)	0.454
ADL disability	295 (10.52%)	184 (22.12%)	35 (7.03%)	50 (11.57%)	26 (2.49%)	<0.001

Values are median (IQR) or n (%)

*Abbreviations*: *ADL* Activities of daily living, *IQR* Inter-quartile range

### Association of changes in frailty status with outcomes

**Fig. 3:**
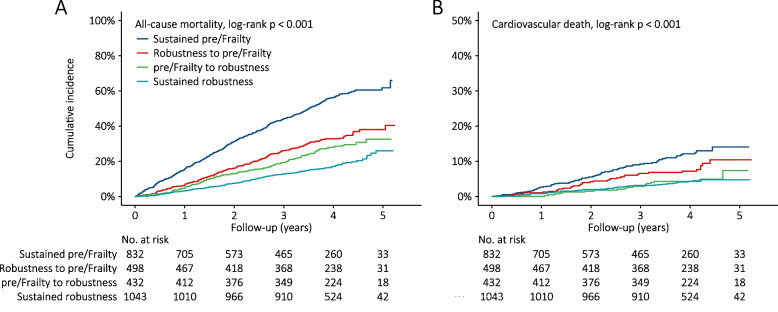
Cumulative incidence plotted by Kaplan-Meier curves. Note: (**A**) incidence of all-cause mortality by changes in frailty status; (**B**) incidence of cardiovascular death by changes in frailty status

During the follow-up of 9530.1 person-years, 952 all-cause mortalities were recorded. Among them, there were 170 deaths attributed to cardiovascular diseases. eTable 5 shows the detailed information about the causes of death. The all-cause mortality rates were 19.8 (95% CI: 18.2-21.4), 9.8 (95% CI: 8.4-11.3), 7.9 (95% CI: 6.6-9.3) and 4.8 (95% CI: 4.2-5.5) per 100 person-years in subgroup of sustained pre/frailty, subgroup of robustness to pre/frailty, subgroup of pre/frailty to robustness and subgroup of sustained robustness, respectively. The corresponding cardiovascular mortality rates 3.1 (95% CI: 2.4-3.8), 2.1 (95% CI: 1.4-2.8), 1.2 (95% CI: 0.6-1.7) and 1.1 (95% CI: 0.7-1.4) per 100 person-years across the four subgroups (Table 2). As shown in Fig. 3, Kaplan-Meier analysis also demonstrated that the cumulative incidence of both all-cause mortality (Log-rank *p* < 0.001) and cardiovascular death (Log-rank *p* < 0.001) were significantly lower in more robust older participants.

**Table 2:** Association of changes in frailty status with cardiovascular death and all-cause mortality

	Sustained pre/Frailty	Robustness to pre/Frailty	pre/Frailty to robustness	Sustained robustness
*All-cause mortality*				
No. of participants (n)	832	498	432	1043
Deaths (n)	473	169	123	187
Follow-up (PYs)	2388.9	1716.6	1553.1	3871.6
Mortality rate (95% CI)^a^	19.8 (18.2-21.4)	9.8 (8.4-11.3)	7.9 (6.6-9.3)	4.8 (4.2-5.5)
Unadjusted HR (95% CI), p	1.00 (ref)	0.49 (0.41-0.58), <0.001	0.39 (0.32-0.48), <0.001	0.24 (0.20-0.28), <0.001
Adjusted HR (95% CI), p				
model 1^b^	1.00 (ref)	0.57 (0.48-0.68), <0.001	0.50 (0.41-0.61), <0.001	0.37 (0.30-0.44), <0.001
model 2^c^	1.00 (ref)	0.61 (0.51-0.73), <0.001	0.51 (0.42-0.63), <0.001	0.41 (0.34-0.49), <0.001
E-value^d^	NA	2.17	2.56	3.09
*Cardiovascular death*				
No. of participants (n)	832	498	432	1043
Deaths (n)	75	36	18	41
Follow-up (PYs)	2388.9	1716.6	1553.1	3871.6
Mortality rate (95% CI)^a^	3.1 (2.4-3.8)	2.1 (1.4-2.8)	1.2 (0.6-1.7)	1.1 (0.7-1.4)
Unadjusted HR (95% CI), p	1.00 (ref)	0.65 (0.44-0.97), 0.037	0.36 (0.21-0.60), <0.001	0.33 (0.22-0.48), <0.001
Adjusted HR (95% CI), p				
model 1^b^	1.00 (ref)	0.75 (0.50-1.12), 0.155	0.45 (0.26-0.75), 0.002	0.48 (0.32-0.72), <0.001
model 2^c^	1.00 (ref)	0.79 (0.52-1.19), 0.256	0.45 (0.26-0.76), 0.003	0.51 (0.33-0.78), 0.002
E-value^d^	NA	1.86	3.87	3.33

*Abbreviations*: *CI* Confidence interval, *HR* Hazard ratio, *PYs* Person-years

^a^per 100 person-years

^b^Adjustment with sex, and age

^c^Adjustment with sex, age, education, marital status, income, residence, living with family, current smoking, current drinking, current exercise, regular intake of foods, comorbidities, and ADL disability

^d^The E-values were for the adjusted HRs from model 2

Table 2 presents the HRs according to the transition in frailty status. Compared with subgroup of sustained pre/frailty, the multivariate-adjusted HRs of all-cause mortality were 0.61 (95% CI: 0.51–0.73, *p* < 0.001), 0.51 (95% CI: 0.42–0.63, *p* < 0.001) and 0.41 (0.34–0.49, *p* < 0.001) in subgroup of robustness to pre/frailty, subgroup of pre/frailty to robustness, and subgroup of sustained robustness. The fully adjusted HRs of cardiovascular death were 0.79 (95% CI: 0.52–1.19, *p* = 0.256), 0.45 (95% CI: 0.26–0.76, *p* = 0.003) and 0.51 (0.33–0.78, *p* = 0.002) in subgroup of robustness to pre/frailty, subgroup of pre/frailty to robustness, and subgroup of sustained robustness when comparing to subgroup of sustained pre/frailty.

### Stratified analysis

For cardiovascular death, in the subgroups by sex, age, education, marital status, residence, living with family, lifestyles, and ADL disability, results of analyses did not change materially with all p_interaction_ ≥ 0.05 (eFigure 1). Similar results were observed for all-cause mortality except in the subgroup analysis by marital status. The decreased risk of all-cause mortality was more pronounced in older participants who were in marriage than older participants who were not in marriage with p_interaction_ = 0.001 (eFigure 2).

### Sensitivity analysis

Extensive sensitivity analyses were conducted. Similar findings were observed when further controlling for frailty status at wave 2014 (eTable 6). When treating participants lost to follow-up as censored at the median or the end of follow-up, the results remained the same (eTable 7 and eTable 8). After excluding deaths within the first year, the results remained unchanged (eTable 9). The association of changes in frailty status with cardiovascular death was similar when accounting for competing risk by non-cardiovascular death (eTable 10). Moreover, when considering only participants without co-morbidities, the association remained consistent (eTable 11). Additionally, the association between changes in frailty status and cardiovascular death remained similar when considering death of unknown reasons as cardiovascular death (eTable 12). Finally, similar results were also observed after multiple imputation (eTable 13). For cardiovascular death, based on model 2 in Table 2, the E-values were 1.86, 3.87 and 3.33 in subgroup of robustness to pre/frailty, subgroup of pre/frailty to robustness and subgroup of sustained robustness, respectively. For all-cause mortality, the corresponding E-values were 2.17, 2.56, 3.09.

### Exploratory analysis

Finally, to expand the main results, we performed exploratory analyses to examine the associations of changes in two out of three frailty status (i.e. frailty, prefrailty, and robustness) with outcomes, and to assess the associations of improved levels of frailty status with outcomes. Due to the relatively small sample size in each group, the results here were considered as exploratory analysis.

When comparing to subgroup of sustained frailty, the risk of cardiovascular death and all-cause mortality were significantly lower in subgroup of sustained prefrailty with adjusted HR at 0.47 (95% CI: 0.23-0.95, *p* = 0.036) and 0.49 (95% CI: 0.37-0.65. *p* < 0.001). Subjects with transition from frailty to prefrailty also had a 27% lower risk of all-cause mortality when compared to those with sustained frailty (adjusted HR: 0.63, 95% CI: 0.47-0.84, *p* = 0.002). No significant difference with regards to all-cause mortality or cardiovascular death was observed in other subgroups (eTable 14).

The adjusted HRs of all-cause mortality were 0.69 (95% CI: 0.50-0.95, *p* = 0.024), 0.39 (95% CI: 0.26-0.58, *p* < 0.001) and 0.25 (95% CI: 0.18-0.33, *p* < 0.001) in subgroup of robustness to frailty, subgroup of frailty to robustness, subgroup of sustained robustness when comparing to subgroup of sustained frailty. For cardiovascular death, subjects with sustained robustness had significantly lower risk in comparison to those with sustained frailty (adjusted HR: 0.39, 95% CI: 0.19-0.79, *p* = 0.009). No significant difference with regards to all-cause mortality or cardiovascular death was observed in other subgroups (eTable 15).

The adjusted HRs of all-cause mortality were 0.63 (95% CI: 0.48-0.82, *p* = 0.001), 0.65 (95% CI: 0.49-0.85, *p* = 0.002) and 0.56 (95% CI: 0.44-0.71, *p* < 0.001) in subgroup of robustness to prefrailty, subgroup of prefrailty to robustness, subgroup of sustained robustness when comparing to subgroup of sustained prefrailty. However, the risk of cardiovascular death showed no significant difference among the 4 groups (eTable 16).

## Discussion

In the present study, we determined the association of changes in frailty status with the risk of all-cause mortality and cardiovascular death in older people. For all-cause mortality, those who transitioned from robustness to pre/frailty had a 39% reduced risk when compared to those who sustained pre/frail, those who transitioned from pre/frailty to robustness had a 49% reduced risk, and those who sustained robust had a 59% reduced risk. Similar trend was found for cardiovascular death. Stratified analysis and extensive sensitivity analyses also supported the robustness of the present findings.

The frailty process is a transitional state in the dynamic progression from robustness to functional decline. In the present study, although majority of older people remained unchanged from their baseline frailty status, both worsen frailty and improved frailty were observed. Previous studies have demonstrated that sociodemographic factors, personal behaviors and clinical factors, such as age, sex, exercise, co-morbidities were significantly involved in the dynamic process [19, 20]. Certain healthy lifestyle behaviors, such as farming, physical exercise, intellectual activity, social participations and maintaining good nutritional status have been shown to contribute to improvement in frailty status in older people [21, 22]. Muscle strength training and protein supplementation were reported as effective intervention to delay or reverse frailty [23]. Given the fact that frailty, which is potentially reversible, and ageing, which is not, early detection and prevention of frailty in older people is of great significance in the era of global aging.

Most of the previous studies reported worsen frailty led to adverse health outcomes. For example, in a previous study using data of 11165 older people from the wave 2002 and 2005 of CLHLS, Liu et al reported that older people with worsen and remaining frail, when defined by frailty index based on 44 health deficits, had significantly higher risk of 3-year painful death, namely ≥ 30 bedridden days with suffering before death when comparing to those with remaining robust [24], indicating that the quality of death is influenced by frailty transitions as well. In a sample of Taiwan population, older people with worsen frailty status, when assessed by Fried’s frailty phenotype, exhibited higher risk of health care utilization and subsequent higher risk of 1-year all-cause mortality [10], suggesting increased medical expenditures due to frailty deterioration. A 6-year prospective study based on 921 community-dwelling older people aged 65-99 years in Taiwan also demonstrated that robust older people with transition to frail status and frail older people remaining in frail status had a 2.76-fold and 4.08-fold increased mortality risk when comparing to those remaining robust [9]. In another cohort consisted of middle-aged and older Taiwanese, increasing frailty index was significantly associated with increased 4-year all-cause mortality and certain cause-specific mortality, including mortality due to infection, malignancy, cardiometabolic/cerebrovascular diseases, organ failure and some others [25]. Using four longitudinal studies of aging consisting of 24961 older respondents aged more than 65 years old, Stolz et al demonstrated that an increase in annual frailty index growth by 0.01 was associated with an increased all-cause mortality risk of HR = 1.56 (95% CI = 1.49–1.63) in the Health and Retirement Study, HR = 1.24 (95% CI = 1.13–1.35) in the Survey of Health, Ageing and Retirement in Europe, HR = 1.40 (95% CI = 1.25–1.52) in the English Longitudinal Survey of Ageing, and HR = 1.71 (95% CI = 1.46–2.01) in the Longitudinal Aging Study Amsterdam [12].

Older people with sustained robustness and improved frailty had significantly lower risk of cardiovascular death in the present study as well. The underlying mechanisms are complicated and multidimensional. Ramsay et al demonstrated that frailty was significantly associated with a range of cardiovascular factors in older British men, such as obesity, high-density lipoprotein cholesterol, hypertension, heart rate, etc [26]. A recent systemic review also indicated that multiple cardiometabolic risk factors, including abdominal obesity, hyperglycemia, dyslipidemia and elevated blood pressure were significantly associated with increased risk of frailty in older people [27]. In older patients with already established cardiovascular disease, such as acute coronary syndrome, heart failure, valvular heart disease, frailty status was independently associated with increased adverse outcomes and mortality [28–30]. In fact, the leading contributors to disease burden in older people are cardiovascular diseases, which accounted for 30.3% of the total burden in people aged 60 years and older [31]. And thus, identification of older people with pre/frailty or worsen frailty who could benefit from frailty intervention in contemporary cardiovascular practice is recommended to optimize cardiovascular care and reduce corresponding disease burden in older people.

Our study has a number of strengths, including longitudinal assessment of frailty status, which conforms to the dynamic nature of frailty, in-depth analysis about the association between frailty transitions and cardiovascular specific death in addition to all-cause mortality, and extensive sensitivity analyses and exploratory analyses to support the main findings. Collaborated with most of the previous studies [9, 10, 12], our results showed that improved frailty and remaining robust is significantly associated with lower risk of all-cause mortality in older Chinese people. In addition, older people with improved frailty or remained robust also had significantly lower risk of cardiovascular death. Actually, there are limited prospective data investigating the impact of improved frailty on health outcomes. In one recently published article, Davis et al demonstrated that frailty remission could potentially lower the risk of future falls in participants aged more than 50 [32]. Wang et al recently showed that intervention of frailty in older patients during hospitalization could improve the ability of ADL and frailty status, shorten the length of hospital stay, lower both medical costs and 1- or 3-month readmission rates [33]. However, a previous meta-analysis indicated that interventions for frail community-dwelling older adults have no significant effect on adverse outcomes, including mortality, institutionalization, accidental falls and hospitalization [34]. The authors explained the negative conclusion with heterogeneity within different studies. Future studies using some realist approaches might provide more information about the effect of frailty intervention.

Nevertheless, several limitations of this study need to be mentioned. First, about a quarter of death causes were unknown. The association between frailty transitions and cause-specific death would provide more useful information. Second, the frailty status was measured twice over a 3-year interval, which may not be adequately to capture the changes timely, especially when some urgent and severe morbidities occur. Third, although many confounders have been adjusted to minimize the effect of potential confounders, other residual confounding is possible. Fourth, the use of self-reported data on components of frailty status and medical conditions may lead to biased results. Last, a relatively small sample size may lead to less power for current results, especially for the exploratory analyses.

## Conclusion

Those with sustained pre/frailty were more likely to be older, female and had high prevalence of ADL disability. Improved frailty and remaining robust is significantly associated with lower risk of all-cause mortality in older people. In addition, older people with improved frailty or remained robust also had significantly lower risk of cardiovascular death. This benefit persisted after accounting for traditional modifiable and non-modifiable cardiovascular risk factors. Frailty is a dynamic process, which is bidirectional and potentially reversal. However, about half the older people had sustained pre/frailty or even had worsen frailty. Therefore, frailty status should be assessed periodically to respond fast if frailty deteriorates. Specific interventions and effective health-care policies to prevent frailty deterioration and reduce its adverse health consequences in older people is urged.

### Supplementary Information

**Additional file 1: eFigure 1.** Stratified analyses by potential modifiers of the association between changes in frailty status and risk of all-cause mortality.

**Additional file 2: eFigure 2.** Stratified analyses by potential modifiers of the association between changes in frailty status and risk of cardiovascular death.

**Additional file 3: eTable 1.** Definitions of baseline variables in the present study.

**Additional file 4: eTable 2.** Distributions of baseline variables with missing data.

**Additional file 5: eTable 3.** Association of frailty status at wave 2011 with cardiovascular death and all-cause mortality.

**Additional file 6: eTable 4.** Association of frailty status at wave 2014 with cardiovascular death and all-cause mortality.

**Additional file 7: eTable 5.** Number of different causes of death.

**Additional file 8: eTable 6.** Association of changes in frailty status with cardiovascular death and all-cause mortality, further controlling for frailty status at wave 2014.

**Additional file 9: eTable 7.** Association of changes in frailty status with cardiovascular death and all-cause mortality, in considering the losses censored at the median time of follow-up (3.88 years).

**Additional file 10: eTable 8.** Association of changes in frailty status with cardiovascular death and all-cause mortality, in considering the losses censored at the end of follow-up (5.24 years).

**Additional file 11: eTable 9.** Association of changes in frailty status with cardiovascular death and all-cause mortality, after excluding deaths within the first year.

**Additional file 12: eTable 10.** Association of changes in frailty status with cardiovascular death, accounting for competing risk by non-cardiovascular death.

**Additional file 13: eTable 11.** Association of changes in frailty status with cardiovascular death and all-cause mortality, after excluding participants with some comorbidities.

**Additional file 14: eTable 12.** Association of changes in frailty status with cardiovascular death, after considering deaths of unknown reasons as cardiovascular death.

**Additional file 15: eTable 13.** Association of changes in frailty status with cardiovascular death and all-cause mortality after multiple imputation^a^.

**Additional file 16: eTable 14.** Association of changes in frailty status with cardiovascular death and all-cause mortality in participants with prefrailty or frailty at waves 2011 and 2014.

**Additional file 17: eTable 15.** Association of changes in frailty status with cardiovascular death and all-cause mortality in participants with robustness or frailty at waves 2011 and 2014.

**Additional file 18: eTable 16.** Association of changes in frailty status with cardiovascular death and all-cause mortality in participants with prefrailty or robustness at waves 2011 and 2014.

## Data Availability

The datasets analyzed during the current study are available in the National Archive of Computerized Data on Aging (NACDA) repository, persistent web link: https://www.icpsr.umich.edu/icpsrweb/NACDA/series/487.
