# Supplementary material for: Association of changes in frailty status with the risk of all-cause mortality and cardiovascular death in older people: results from the Chinese Longitudinal Healthy Longevity Survey (CLHLS)
Source: BMC Geriatr. 2024 Jan 25;24:96. doi: 10.1186/s12877-024-04682-2 (PMC10809745; doi:10.1186/s12877-024-04682-2)

Changes in frailty status	Deaths (%)	Adjusted HR (95% CI)	Deaths (%)	Adjusted HR (95% CI)	p for interaction
	Sex: male		Sex: female		
Sustained pre/Frailty	191 (62.42%)	1.00 (ref)	282 (53.61%)	1.00 (ref)	
Robustness to pre/Frailty	94 (39.17%)	0.57 (0.44-0.74)	75 (29.07%)	0.65 (0.50-0.84)	
pre/Frailty to robustness	64 (31.37%)	0.47 (0.35-0.63)	59 (25.88%)	0.54 (0.41-0.73)	0.753
Sustained robustness	124 (20.00%)	0.36 (0.28-0.47)	63 (14.89%)	0.47 (0.35-0.63)	
	Age: <80 years		Age: ≥80 years		
Sustained pre/Frailty	70 (29.05%)	1.00 (ref)	403 (68.19%)	1.00 (ref)	
Robustness to pre/Frailty	38 (17.35%)	0.59 (0.39-0.89)	131 (46.95%)	0.58 (0.48-0.71)	
pre/Frailty to robustness	26 (12.32%)	0.37 (0.24-0.59)	97 (43.89%)	0.49 (0.39-0.61)	0.822
Sustained robustness	80 (11.28%)	0.37 (0.26-0.53)	107 (32.04%)	0.38 (0.30-0.47)	
	Education: no school		Education: 1 year or more		
Sustained pre/Frailty	310 (58.49%)	1.00 (ref)	163 (53.97%)	1.00 (ref)	
Robustness to pre/Frailty	99 (35.61%)	0.65 (0.51-0.82)	70 (31.82%)	0.54 (0.41-0.73)	
pre/Frailty to robustness	73 (33.03%)	0.59 (0.45-0.77)	50 (23.70%)	0.41 (0.29-0.57)	0.117
Sustained robustness	86 (21.23%)	0.50 (0.38-0.65)	101 (15.83%)	0.33 (0.25-0.43)	
	Marital status: not in marriage		Marital status: in marriage		
Sustained pre/Frailty	328 (62.12%)	1.00 (ref)	145 (47.70%)	1.00 (ref)	
Robustness to pre/Frailty	106 (39.55%)	0.66 (0.53-0.83)	63 (27.39%)	0.49 (0.36-0.66)	
pre/Frailty to robustness	84 (37.67%)	0.62 (0.49-0.80)	39 (18.66%)	0.32 (0.22-0.45)	0.001
Sustained robustness	99 (25.52%)	0.51 (0.40-0.65)	88 (13.44%)	0.29 (0.22-0.39)	
	Income: fair/poor		Income: rich		
Sustained pre/Frailty	388 (55.75%)	1.00 (ref)	85 (62.50%)	1.00 (ref)	
Robustness to pre/Frailty	130 (32.42%)	0.60 (0.49-0.73)	39 (40.21%)	0.62 (0.40-0.94)	
pre/Frailty to robustness	97 (27.17%)	0.50 (0.40-0.63)	26 (34.67%)	0.53 (0.33-0.85)	0.951
Sustained robustness	144 (17.93%)	0.41 (0.33-0.51)	43 (17.92%)	0.36 (0.24-0.55)	
	Residence: rural		Residence: urban		
Sustained pre/Frailty	278 (55.05%)	1.00 (ref)	195 (59.63%)	1.00 (ref)	
Robustness to pre/Frailty	91 (35.41%)	0.71 (0.55-0.91)	78 (32.37%)	0.50 (0.38-0.65)	
pre/Frailty to robustness	62 (25.83%)	0.50 (0.37-0.66)	61 (31.77%)	0.51 (0.38-0.68)	0.172
Sustained robustness	91 (16.88%)	0.44 (0.34-0.57)	96 (19.05%)	0.36 (0.27-0.47)	
	Living with family: no		Living with family: yes		
Sustained pre/Frailty	80 (49.69%)	1.00 (ref)	393 (58.57%)	1.00 (ref)	
Robustness to pre/Frailty	27 (25.23%)	0.65 (0.41-1.04)	142 (36.32%)	0.60 (0.49-0.73)	
pre/Frailty to robustness	19 (25.68%)	0.54 (0.33-0.91)	104 (29.05%)	0.50 (0.40-0.63)	0.881
Sustained robustness	35 (18.42%)	0.52 (0.33-0.80)	152 (17.82%)	0.39 (0.32-0.48)	
	Lifestyle: unhealthy		Lifestyle: healthy		
Sustained pre/Frailty	446 (56.74%)	1.00 (ref)	27 (58.70%)	1.00 (ref)	
Robustness to pre/Frailty	152 (33.19%)	0.60 (0.50-0.73)	17 (42.50%)	0.59 (0.29-1.19)	
pre/Frailty to robustness	116 (29.00%)	0.52 (0.42-0.64)	7 (21.88%)	0.39 (0.15-1.03)	0.416
Sustained robustness	171 (18.47%)	0.42 (0.34-0.51)	16 (13.68%)	0.21 (0.10-0.47)	
	ADL disability: no		ADL disability: yes		
Sustained pre/Frailty	328 (50.62%)	1.00 (ref)	145 (78.80%)	1.00 (ref)	
Robustness to pre/Frailty	154 (33.26%)	0.66 (0.54-0.80)	15 (42.86%)	0.41 (0.23-0.74)	
pre/Frailty to robustness	102 (26.70%)	0.55 (0.44-0.69)	21 (42.00%)	0.34 (0.21-0.56)	0.421
Sustained robustness	177 (17.40%)	0.44 (0.36-0.54)	10 (38.46%)	0.30 (0.15-0.59)	

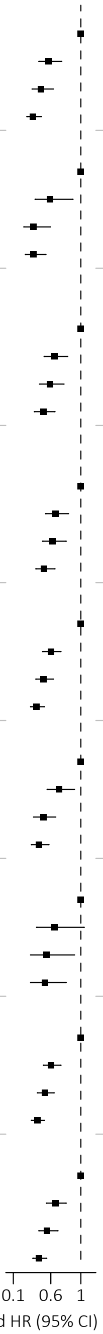

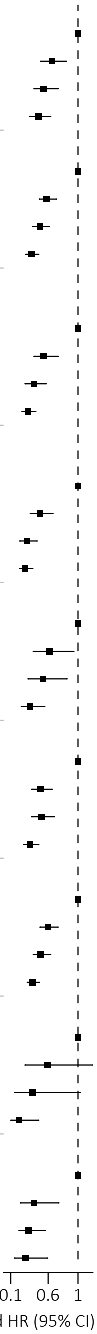

Supplement: Supplementary file 1 — Additional file 1: eFigure 1. Stratified analyses by potential modifiers of the association between changes in frailty status and risk of all-cause mortality. [file 12877_2024_4682_MOESM1_ESM.pdf]
