# Supplementary material for: Association of changes in frailty status with the risk of all-cause mortality and cardiovascular death in older people: results from the Chinese Longitudinal Healthy Longevity Survey (CLHLS)
Source: BMC Geriatr. 2024 Jan 25;24:96. doi: 10.1186/s12877-024-04682-2 (PMC10809745; doi:10.1186/s12877-024-04682-2)

Changes in frailty status	Deaths (%)	Adjusted HR (95% CI)	Deaths (%)	Adjusted HR (95% CI)	p for interaction
	Sex: male		Sex: female		
Sustained pre/Frailty	32 (10.46%)	1.00 (ref)	43 (8.17%)	1.00 (ref)	
Robustness to pre/Frailty	25 (10.42%)	0.86 (0.50-1.49)	11 (4.26%)	0.60 (0.30-1.20)	0.105
pre/Frailty to robustness	8 (3.92%)	0.29 (0.13-0.65)	10 (4.39%)	0.65 (0.32-1.33)	
Sustained robustness	24 (3.87%)	0.34 (0.19-0.60)	17 (4.02%)	0.88 (0.47-1.67)	
	Age: <80 years		Age: ≥80 years		
Sustained pre/Frailty	14 (5.81%)	1.00 (ref)	61 (10.32%)	1.00 (ref)	
Robustness to pre/Frailty	9 (4.11%)	0.76 (0.31-1.82)	27 (9.68%)	0.79 (0.49-1.26)	0.952
pre/Frailty to robustness	5 (2.37%)	0.38 (0.13-1.08)	13 (5.88%)	0.41 (0.22-0.76)	
Sustained robustness	18 (2.54%)	0.40 (0.19-0.86)	23 (6.89%)	0.50 (0.30-0.84)	
	Education: no school		Education: 1 year or more		
Sustained pre/Frailty	47 (8.87%)	1.00 (ref)	28 (9.27%)	1.00 (ref)	
Robustness to pre/Frailty	22 (7.91%)	0.95 (0.56-1.63)	14 (6.36%)	0.59 (0.30-1.16)	0.280
pre/Frailty to robustness	11 (4.98%)	0.58 (0.29-1.14)	7 (3.32%)	0.28 (0.12-0.66)	
Sustained robustness	20 (4.94%)	0.74 (0.42-1.33)	21 (3.29%)	0.31 (0.16-0.58)	
	Marital status: not in marriage		Marital status: in marriage		
Sustained pre/Frailty	47 (8.90%)	1.00 (ref)	28 (9.21%)	1.00 (ref)	
Robustness to pre/Frailty	22 (8.21%)	0.95 (0.55-1.62)	14 (6.09%)	0.54 (0.28-1.06)	0.258
pre/Frailty to robustness	11 (4.93%)	0.57 (0.29-1.12)	7 (3.35%)	0.26 (0.11-0.61)	
Sustained robustness	19 (4.90%)	0.72 (0.40-1.31)	22 (3.36%)	0.34 (0.18-0.64)	
	Income: fair/poor		Income: rich		
Sustained pre/Frailty	64 (9.20%)	1.00 (ref)	11 (8.09%)	1.00 (ref)	
Robustness to pre/Frailty	25 (6.23%)	0.69 (0.43-1.11)	11 (11.34%)	1.50 (0.56-4.02)	0.594
pre/Frailty to robustness	14 (3.92%)	0.42 (0.23-0.75)	4 (5.33%)	0.78 (0.22-2.67)	
Sustained robustness	33 (4.11%)	0.50 (0.31-0.81)	8 (3.33%)	0.45 (0.16-1.32)	
	Residence: rural		Residence: urban		
Sustained pre/Frailty	44 (8.71%)	1.00 (ref)	31 (9.48%)	1.00 (ref)	
Robustness to pre/Frailty	22 (8.56%)	1.06 (0.61-1.83)	14 (5.81%)	0.53 (0.28-1.03)	0.285
pre/Frailty to robustness	13 (5.42%)	0.62 (0.33-1.19)	5 (2.60%)	0.26 (0.10-0.69)	
Sustained robustness	20 (3.71%)	0.51 (0.27-0.94)	21 (4.17%)	0.47 (0.25-0.87)	
	Living with family: no		Living with family: yes		
Sustained pre/Frailty	11 (6.83%)	1.00 (ref)	64 (9.54%)	1.00 (ref)	
Robustness to pre/Frailty	9 (8.41%)	1.19 (0.45-3.19)	27 (6.91%)	0.71 (0.44-1.14)	0.663
pre/Frailty to robustness	3 (4.05%)	0.45 (0.11-1.87)	15 (4.19%)	0.43 (0.24-0.77)	
Sustained robustness	6 (3.16%)	0.46 (0.14-1.47)	35 (4.10%)	0.51 (0.32-0.81)	
	Lifestyle: unhealthy		Lifestyle: healthy		
Sustained pre/Frailty	71 (9.03%)	1.00 (ref)	4 (8.70%)	1.00 (ref)	
Robustness to pre/Frailty	34 (7.42%)	0.81 (0.53-1.25)	2 (5.00%)	0.65 (0.10-4.38)	0.907
pre/Frailty to robustness	16 (4.00%)	0.44 (0.25-0.76)	2 (6.25%)	0.59 (0.08-4.28)	
Sustained robustness	37 (4.00%)	0.53 (0.34-0.82)	4 (3.42%)	0.18 (0.03-1.05)	
	ADL disability: no		ADL disability: yes		
Sustained pre/Frailty	51 (7.87%)	1.00 (ref)	24 (13.04%)	1.00 (ref)	
Robustness to pre/Frailty	32 (6.91%)	0.83 (0.53-1.31)	4 (11.43%)	0.81 (0.22-2.92)	0.842
pre/Frailty to robustness	16 (4.19%)	0.52 (0.29-0.92)	2 (4.00%)	0.12 (0.02-0.66)	
Sustained robustness	39 (3.83%)	0.56 (0.35-0.89)	2 (7.69%)	0.34 (0.07-1.77)	

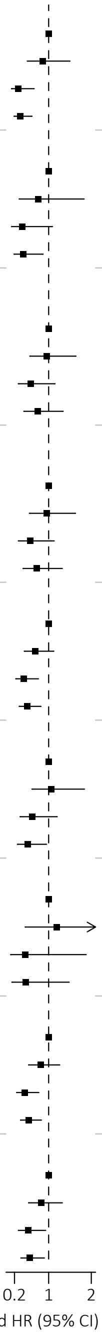

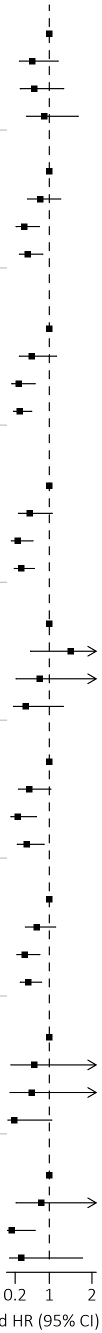

Supplement: Supplementary file 2 — Additional file 2: eFigure 2. Stratified analyses by potential modifiers of the association between changes in frailty status and risk of cardiovascular death. [file 12877_2024_4682_MOESM2_ESM.pdf]
