# Supplementary material for: Association of changes in frailty status with the risk of all-cause mortality and cardiovascular death in older people: results from the Chinese Longitudinal Healthy Longevity Survey (CLHLS)
Source: BMC Geriatr. 2024 Jan 25;24:96. doi: 10.1186/s12877-024-04682-2 (PMC10809745; doi:10.1186/s12877-024-04682-2)
Supplement: Supplementary file 3 — Additional file 3: eTable 1. Definitions of baseline variables in the present study. [file 12877_2024_4682_MOESM3_ESM.docx]

eTable 1. Definitions of baseline variables in the present study

| Variable in the present study | Components  of variable | Questions in the CLHLS questionnaire | Options in the CLHLS questionnaire | Scales of reclassification  in the present study |
| --- | --- | --- | --- | --- |
| Sex |  |  | • male • female | • Male • Female |
| Age |  |  |  | • Continuous (years) |
| Education |  | How many years did you attend school? | • years of school • don't know • missing | • No school: years of school = 0 • 1 year or more: years of school >= 1 • missing: don't know, missing |
| Marital status |  | Current marital status? | • currently married and living with spouse • separated • divorced • widowed • never married • don't know • missing | • In marriage: currently married and living with spouse, separated • Not in marriage: divorced, widowed, never married • missing: don't know, missing |
| Income |  | How do you rate your economic status compared with others in your local area? | • very rich • rich  • so so • poor  • very poor  • didn’t answer • missing | • Rich: very rich, rich • Fair/Poor: so so, poor, very poor • missing: didn`t answer, missing |
| Residence |  | Current residence area of interviewee? | • city • town  • rural | • Urban: city, town • Rural: rural |
| Living with family |  | Co-residence? | • with household member(s) • alone • in an institution • missing | • Yes: with household member(s) • No: alone, in an institution • missing |
| Current smoking |  | Do you smoke at present? | • yes • no • missing | • Yes: yes • No: no • missing: missing |
| Current drinking |  | Do you drink at present? | • yes • no • don't know • missing | • Yes: yes • No: no • missing: don't know, missing |
| Current exercise |  | Do you do exercises regularly at present? | • yes • no • don't know • missing | • Yes: yes • No: no • missing: don't know, missing |
| Regular intake of fruits, and vegetables, respectively |  | Do you eat these foods, respectively? | • almost everyday • except winter/quite often • occasionally • rarely or never • don't know • missing | • Regular intake: almost everyday, except winter/quite often • No regular intake: occasionally, rarely or never • missing: don't know, missing |
| Regular intake of meats, fishes, eggs, and beans, respectively |  | Do you eat these foods, respectively? | • almost everyday • not everyday, but at least once per week • not every week, but at least once per month • not every month, but occasionally • rarely or never • don't know • missing | • Regular intake: almost everyday; not everyday, but at least once per week • No regular intake: not every week, but at least once per month; not every month, but occasionally; rarely or never • missing: don't know, missing |
| Hypertension, diabetes, heart diseases, cerebrovascular diseases, respiratory diseases, and cancer, respectively |  | Are you suffering from these diseases, respectively? | • yes • no • don't know • missing | • Yes: yes • No: no • missing: don't know, missing |
| ADL disability | Bathing | Without assistance? | • without assistance • one part assistance • more than one part assistance • don't know • missing | • In the CLHLS survey, six items of daily self-care ability were collected from each participant based on the Katz index: dressing, bathing, transferring, toileting, continence, and eating. Each item included three answers: complete independence, partially dependence, and complete dependence.   ADL disability was defined as present if participants needed any assistance in performing at least one of the six self-care activities. • missing: don't know, missing |
|  | Dressing | Get clothes and get completely dressed without assistance? | • without assistance • need assistance for trying shoes • assistance in getting clothes and getting dressed • missing |  |
|  | Toileting | Go to the toilet, cleans self, and arranges clothes without assistance (may use object for support such as cane, walker, or wheelchair)? | • without assistance • assistance in cleaning or arranging clothes • don't use toilet • missing |  |
|  | Transferring | Get in and out of bed as well as in and out of a chair without assistance (may use object for support such as cane or walker)? | • without assistance • with assistance • bedridden • missing |  |
|  | Continence | Has complete control of urination and bowel movement without assistance? | • without assistance • occasional accidents • incontinent • missing |  |
|  | Feeding | Feed self without assistance? | • without assistance • with some help • need feeding • missing |  |

More detailed information about these covariates can be found on: <https://agingcenter.duke.edu/CLHLS>. Abbreviations: ADL = activities of daily living, CLHLS = Chinese Longitudinal Healthy Longevity Survey.
