# Supplementary material for: Association of changes in frailty status with the risk of all-cause mortality and cardiovascular death in older people: results from the Chinese Longitudinal Healthy Longevity Survey (CLHLS)
Source: BMC Geriatr. 2024 Jan 25;24:96. doi: 10.1186/s12877-024-04682-2 (PMC10809745; doi:10.1186/s12877-024-04682-2)
Supplement: Supplementary file 4 — Additional file 4: eTable 2. Distributions of baseline variables with missing data. [file 12877_2024_4682_MOESM4_ESM.docx]

eTable 2. Distributions of baseline variables with missing data

| Variable | Number of  missing data | Percentage of missing data (%) |
| --- | --- | --- |
| Sex | 0 | 0.00 |
| Age | 0 | 0.00 |
| Education | 12 | 0.29 |
| Marital status | 12 | 0.29 |
| Income | 23 | 0.55 |
| Residence | 0 | 0.00 |
| Living with family | 32 | 0.77 |
| Current smoking | 16 | 0.38 |
| Current drinking | 45 | 1.08 |
| Current exercise | 42 | 1.01 |
| Regular intake of foods |  |  |
| Fruits | 4 | 0.10 |
| Vegetables | 9 | 0.22 |
| Meats | 6 | 0.14 |
| Fishes | 4 | 0.10 |
| Eggs | 6 | 0.14 |
| Beans | 9 | 0.22 |
| Comorbidities |  |  |
| Hypertension | 152 | 3.64 |
| Diabetes | 188 | 4.51 |
| Heart diseases | 178 | 4.27 |
| Cerebrovascular diseases | 168 | 4.03 |
| Respiratory diseases | 155 | 3.72 |
| Cancer | 202 | 4.84 |
| ADL disability | 97 | 2.33 |

Participants with missing values were deleted in the main statistical analyses, and we performed multiple imputation for missing values as a sensitivity analysis.

Abbreviations: ADL = activities of daily living.
