# Supplementary material for: Association of changes in frailty status with the risk of all-cause mortality and cardiovascular death in older people: results from the Chinese Longitudinal Healthy Longevity Survey (CLHLS)
Source: BMC Geriatr. 2024 Jan 25;24:96. doi: 10.1186/s12877-024-04682-2 (PMC10809745; doi:10.1186/s12877-024-04682-2)
Supplement: Supplementary file 5 — Additional file 5: eTable 3. Association of frailty status at wave 2011 with cardiovascular death and all-cause mortality. [file 12877_2024_4682_MOESM5_ESM.docx]

eTable 3. Association of frailty status at wave 2011 with cardiovascular death and all-cause mortality

|  | Robustness | Prefrailty | Frailty |
| --- | --- | --- | --- |
| *All-cause mortality* |  |  |  |
| No. of participants (n) | 1541 | 872 | 392 |
| Deaths (n) | 356 | 355 | 241 |
| Follow-up (PYs) | 5588.2 | 2863.3 | 1078.7 |
| Mortality rate (95% CI)^a^ | 6.4 (5.7-7) | 12.4 (11.2-13.6) | 22.3 (19.9-24.8) |
| Adjusted HR (95% CI)^b^, p | 1.00 (ref) | 1.53 (1.32-1.79), <0.001 | 1.99 (1.65-2.41), <0.001 |
|  |  |  |  |
| *Cardiovascular death* |  |  |  |
| No. of participants (n) | 1541 | 872 | 392 |
| Deaths (n) | 77 | 56 | 37 |
| Follow-up (PYs) | 5588.2 | 2863.3 | 1078.7 |
| Mortality rate (95% CI)^a^ | 1.4 (1.1-1.7) | 2 (1.4-2.5) | 3.4 (2.3-4.5) |
| Adjusted HR (95% CI)^b^, p | 1.00 (ref) | 1.15 (0.80-1.64), 0.458 | 1.57 (0.99-2.47), 0.054 |

^a^ per 100 person-years.

^b^ Adjustment with sex, age, education, marital status, income, residence, living with family, current smoking, current drinking, current exercise, regular intake of foods, comorbidities, and ADL disability.

Abbreviations: CI = confidence interval; HR = hazard ratio; PYs = person-years.
