# Supplementary material for: Association of changes in frailty status with the risk of all-cause mortality and cardiovascular death in older people: results from the Chinese Longitudinal Healthy Longevity Survey (CLHLS)
Source: BMC Geriatr. 2024 Jan 25;24:96. doi: 10.1186/s12877-024-04682-2 (PMC10809745; doi:10.1186/s12877-024-04682-2)
Supplement: Supplementary file 6 — Additional file 6: eTable 4. Association of frailty status at wave 2014 with cardiovascular death and all-cause mortality. [file 12877_2024_4682_MOESM6_ESM.docx]

eTable 4. Association of frailty status at wave 2014 with cardiovascular death and all-cause mortality

|  | Robustness | Prefrailty | Frailty |
| --- | --- | --- | --- |
| *All-cause mortality* |  |  |  |
| No. of participants (n) | 1475 | 840 | 490 |
| Deaths (n) | 310 | 307 | 335 |
| Follow-up (PYs) | 5424.7 | 2847.2 | 1258.3 |
| Mortality rate (95% CI)^a^ | 5.7 (5.1-6.3) | 10.8 (9.6-11.9) | 26.6 (24.2-29.1) |
| Adjusted HR (95% CI)^b^, p | 1.00 (ref) | 1.48 (1.26-1.74), <0.001 | 2.74 (2.31-3.27), <0.001 |
|  |  |  |  |
| *Cardiovascular death* |  |  |  |
| No. of participants (n) | 1475 | 840 | 490 |
| Deaths (n) | 59 | 54 | 57 |
| Follow-up (PYs) | 5424.7 | 2847.2 | 1258.3 |
| Mortality rate (95% CI)^a^ | 1.1 (0.8-1.4) | 1.9 (1.4-2.4) | 4.5 (3.4-5.7) |
| Adjusted HR (95% CI)^b^, p | 1.00 (ref) | 1.49 (1.01-2.18), 0.042 | 2.79 (1.84-4.22), <0.001 |

^a^ per 100 person-years.

^b^ Adjustment with sex, age, education, marital status, income, residence, living with family, current smoking, current drinking, current exercise, regular intake of foods, comorbidities, and ADL disability.

Abbreviations: CI = confidence interval; HR = hazard ratio; PYs = person-years.
