# Supplementary material for: Association of changes in frailty status with the risk of all-cause mortality and cardiovascular death in older people: results from the Chinese Longitudinal Healthy Longevity Survey (CLHLS)
Source: BMC Geriatr. 2024 Jan 25;24:96. doi: 10.1186/s12877-024-04682-2 (PMC10809745; doi:10.1186/s12877-024-04682-2)
Supplement: Supplementary file 7 — Additional file 7: eTable 5. Number of different causes of death. [file 12877_2024_4682_MOESM7_ESM.docx]

eTable 5. Number of different causes of death

| Causes of death | Frequency |
| --- | --- |
| Infectious and parasitic diseases | 1 |
| Tumor | 64 |
| Blood, hematopoietic organs and immune diseases | 17 |
| Endocrine, nutritional and metabolic diseases | 14 |
| Mental and behavioral disorders | 5 |
| Neurological diseases | 25 |
| Eye and appendage diseases | 1 |
| Cardiovascular diseases | 170 |
| Respiratory diseases | 95 |
| Diseases of digestive system | 24 |
| Skin and subcutaneous tissue diseases | 2 |
| Musculoskeletal system and connective tissue diseases | 9 |
| Diseases of urogenital system | 12 |
| Injury, poisoning, accident or other external causes | 61 |
| Other causes | 215 |
| Unknown causes | 237 |
