# Supplementary material for: Association of changes in frailty status with the risk of all-cause mortality and cardiovascular death in older people: results from the Chinese Longitudinal Healthy Longevity Survey (CLHLS)
Source: BMC Geriatr. 2024 Jan 25;24:96. doi: 10.1186/s12877-024-04682-2 (PMC10809745; doi:10.1186/s12877-024-04682-2)
Supplement: Supplementary file 8 — Additional file 8: eTable 6. Association of changes in frailty status with cardiovascular death and all-cause mortality, further controlling for frailty status at wave 2014. [file 12877_2024_4682_MOESM8_ESM.docx]

eTable 6. Association of changes in frailty status with cardiovascular death and all-cause mortality, further controlling for frailty status at wave 2014

|  | Sustained pre/Frailty | Robustness to pre/Frailty | pre/Frailty to robustness | Sustained robustness |
| --- | --- | --- | --- | --- |
| *All-cause mortality* |  |  |  |  |
| No. of participants (n) | 832 | 498 | 432 | 1043 |
| Deaths (n) | 473 | 169 | 123 | 187 |
| Follow-up (PYs) | 2388.9 | 1716.6 | 1553.1 | 3871.6 |
| Mortality rate (95% CI)^a^ | 19.8 (18.2-21.4) | 9.8 (8.4-11.3) | 7.9 (6.6-9.3) | 4.8 (4.2-5.5) |
| Adjusted HR (95% CI)^b^, p | 1.00 (ref) | 0.67 (0.56-0.81), <0.001 | 0.38 (0.30-0.47), <0.001 | 0.29 (0.24-0.36), <0.001 |
|  |  |  |  |  |
| *Cardiovascular death* |  |  |  |  |
| No. of participants (n) | 832 | 498 | 432 | 1043 |
| Deaths (n) | 75 | 36 | 18 | 41 |
| Follow-up (PYs) | 2388.9 | 1716.6 | 1553.1 | 3871.6 |
| Mortality rate (95% CI)^a^ | 3.1 (2.4-3.8) | 2.1 (1.4-2.8) | 1.2 (0.6-1.7) | 1.1 (0.7-1.4) |
| Adjusted HR (95% CI)^b^, p | 1.00 (ref) | 0.87 (0.57-1.32), 0.512 | 0.32 (0.18-0.57), <0.001 | 0.35 (0.22-0.58), <0.001 |

^a^ per 100 person-years.

^b^ Adjustment with sex, age, education, marital status, income, residence, living with family, current smoking, current drinking, current exercise, regular intake of foods, comorbidities, ADL disability, and frailty status at wave 2014.

Abbreviations: CI = confidence interval; HR = hazard ratio; PYs = person-years.
