# Supplementary material for: Association of changes in frailty status with the risk of all-cause mortality and cardiovascular death in older people: results from the Chinese Longitudinal Healthy Longevity Survey (CLHLS)
Source: BMC Geriatr. 2024 Jan 25;24:96. doi: 10.1186/s12877-024-04682-2 (PMC10809745; doi:10.1186/s12877-024-04682-2)
Supplement: Supplementary file 9 — Additional file 9: eTable 7. Association of changes in frailty status with cardiovascular death and all-cause mortality, in considering the losses censored at the median time of follow-up (3.88 years). [file 12877_2024_4682_MOESM9_ESM.docx]

eTable 7. Association of changes in frailty status with cardiovascular death and all-cause mortality, in considering the losses censored at the median time of follow-up (3.88 years)

|  | Sustained pre/Frailty | Robustness to pre/Frailty | pre/Frailty to robustness | Sustained robustness |
| --- | --- | --- | --- | --- |
| *All-cause mortality* |  |  |  |  |
| No. of participants (n) | 1044 | 670 | 539 | 1320 |
| Deaths (n) | 473 | 169 | 123 | 187 |
| Follow-up (PYs) | 3211.4 | 2384.0 | 1968.2 | 4946.4 |
| Mortality rate (95% CI)^a^ | 14.7 (13.5-16.0) | 7.1 (6.1-8.1) | 6.2 (5.2-7.3) | 3.8 (3.2-4.3) |
| Adjusted HR (95% CI)^b^, p | 1.00 (ref) | 0.59 (0.49-0.71), <0.001 | 0.55 (0.45-0.68), <0.001 | 0.43 (0.36-0.52), <0.001 |
|  |  |  |  |  |
| *Cardiovascular death* |  |  |  |  |
| No. of participants (n) | 1044 | 670 | 539 | 1320 |
| Deaths (n) | 75 | 36 | 18 | 41 |
| Follow-up (PYs) | 3211.4 | 2384.0 | 1968.2 | 4946.4 |
| Mortality rate (95% CI)^a^ | 2.3 (1.8-2.9) | 1.5 (1.0-2.0) | 0.9 (0.5-1.3) | 0.8 (0.6-1.1) |
| Adjusted HR (95% CI)^b^, p | 1.00 (ref) | 0.76 (0.50-1.14), 0.187 | 0.49 (0.29-0.82), 0.007 | 0.54 (0.35-0.83), 0.005 |

^a^ per 100 person-years.

^b^ Adjustment with sex, age, education, marital status, income, residence, living with family, current smoking, current drinking, current exercise, regular intake of foods, comorbidities, and ADL disability.

Abbreviations: CI = confidence interval; HR = hazard ratio; PYs = person-years.
