# Supplementary material for: Association of changes in frailty status with the risk of all-cause mortality and cardiovascular death in older people: results from the Chinese Longitudinal Healthy Longevity Survey (CLHLS)
Source: BMC Geriatr. 2024 Jan 25;24:96. doi: 10.1186/s12877-024-04682-2 (PMC10809745; doi:10.1186/s12877-024-04682-2)
Supplement: Supplementary file 10 — Additional file 10: eTable 8. Association of changes in frailty status with cardiovascular death and all-cause mortality, in considering the losses censored at the end of follow-up (5.24 years). [file 12877_2024_4682_MOESM10_ESM.docx]

eTable 8. Association of changes in frailty status with cardiovascular death and all-cause mortality, in considering the losses censored at the end of follow-up (5.24 years)

|  | Sustained pre/Frailty | Robustness to pre/Frailty | pre/Frailty to robustness | Sustained robustness |
| --- | --- | --- | --- | --- |
| *All-cause mortality* |  |  |  |  |
| No. of participants (n) | 1044 | 670 | 539 | 1320 |
| Deaths (n) | 473 | 169 | 123 | 187 |
| Follow-up (PYs) | 3499.7 | 2617.9 | 2113.7 | 5323.1 |
| Mortality rate (95% CI)^a^ | 13.5 (12.4-14.6) | 6.5 (5.5-7.4) | 5.8 (4.8-6.8) | 3.5 (3.0-4.0) |
| Adjusted HR (95% CI)^b^, p | 1.00 (ref) | 0.59 (0.50-0.71), <0.001 | 0.56 (0.46-0.69), <0.001 | 0.43 (0.36-0.52), <0.001 |
|  |  |  |  |  |
| *Cardiovascular death* |  |  |  |  |
| No. of participants (n) | 1044 | 670 | 539 | 1320 |
| Deaths (n) | 75 | 36 | 18 | 41 |
| Follow-up (PYs) | 3499.7 | 2617.9 | 2113.7 | 5323.1 |
| Mortality rate (95% CI)^a^ | 2.1 (1.7-2.6) | 1.4 (0.9-1.8) | 0.9 (0.5-1.2) | 0.8 (0.5-1.0) |
| Adjusted HR (95% CI)^b^, p | 1.00 (ref) | 0.76 (0.50-1.15), 0.195 | 0.50 (0.29-0.84), 0.010 | 0.55 (0.36-0.84), 0.006 |

^a^ per 100 person-years.

^b^ Adjustment with sex, age, education, marital status, income, residence, living with family, current smoking, current drinking, current exercise, regular intake of foods, comorbidities, and ADL disability.

Abbreviations: CI = confidence interval; HR = hazard ratio; PYs = person-years.
