# Supplementary material for: Association of changes in frailty status with the risk of all-cause mortality and cardiovascular death in older people: results from the Chinese Longitudinal Healthy Longevity Survey (CLHLS)
Source: BMC Geriatr. 2024 Jan 25;24:96. doi: 10.1186/s12877-024-04682-2 (PMC10809745; doi:10.1186/s12877-024-04682-2)
Supplement: Supplementary file 12 — Additional file 12: eTable 10. Association of changes in frailty status with cardiovascular death, accounting for competing risk by non-cardiovascular death. [file 12877_2024_4682_MOESM12_ESM.docx]

eTable 10. Association of changes in frailty status with cardiovascular death, accounting for competing risk by non-cardiovascular death

|  | Sustained pre/Frailty | Robustness to pre/Frailty | pre/Frailty to robustness | Sustained robustness |
| --- | --- | --- | --- | --- |
| No. of participants (n) | 832 | 498 | 432 | 1043 |
| cardiovascular death (n) | 75 | 36 | 18 | 41 |
| non-cardiovascular death (n)^a^ | 398 | 133 | 105 | 146 |
| Adjusted HR (95% CI)^b^, p | 1.00 (ref) | 0.93 (0.61-1.41), 0.73 | 0.55 (0.32-0.94), 0.028 | 0.63 (0.4-0.99), 0.047 |

^a^ non-cardiovascular death coded as event of competing risk.

^b^ Adjustment with sex, age, education, marital status, income, residence, living with family, current smoking, current drinking, current exercise, regular intake of foods, comorbidities, and ADL disability.

Abbreviations: CI = confidence interval; HR = hazard ratio; PYs = person-years.
