# Supplementary material for: Association of changes in frailty status with the risk of all-cause mortality and cardiovascular death in older people: results from the Chinese Longitudinal Healthy Longevity Survey (CLHLS)
Source: BMC Geriatr. 2024 Jan 25;24:96. doi: 10.1186/s12877-024-04682-2 (PMC10809745; doi:10.1186/s12877-024-04682-2)
Supplement: Supplementary file 13 — Additional file 13: eTable 11. Association of changes in frailty status with cardiovascular death and all-cause mortality, after excluding participants with some comorbidities. [file 12877_2024_4682_MOESM13_ESM.docx]

eTable 11. Association of changes in frailty status with cardiovascular death and all-cause mortality, after excluding participants with some comorbidities

|  | Sustained pre/Frailty | Robustness to pre/Frailty | pre/Frailty to robustness | Sustained robustness |
| --- | --- | --- | --- | --- |
| *All-cause mortality^a^* |  |  |  |  |
| No. of participants (n) | 832 | 498 | 432 | 1043 |
| Deaths (n) | 473 | 169 | 123 | 187 |
| Follow-up (PYs) | 2388.9 | 1716.6 | 1553.1 | 3871.6 |
| Mortality rate (95% CI)^b^ | 19.8 (18.2-21.4) | 9.8 (8.4-11.3) | 7.9 (6.6-9.3) | 4.8 (4.2-5.5) |
| Adjusted HR (95% CI)^c^, p | 1.00 (ref) | 0.60 (0.50-0.73), <0.001 | 0.51 (0.42-0.63), <0.001 | 0.40 (0.33-0.49), <0.001 |
|  |  |  |  |  |
| *Cardiovascular death^d^* |  |  |  |  |
| No. of participants (n) | 832 | 498 | 432 | 1043 |
| Deaths (n) | 75 | 36 | 18 | 41 |
| Follow-up (PYs) | 2388.9 | 1716.6 | 1553.1 | 3871.6 |
| Mortality rate (95% CI)^b^ | 3.1 (2.4-3.8) | 2.1 (1.4-2.8) | 1.2 (0.6-1.7) | 1.1 (0.7-1.4) |
| Adjusted HR (95% CI)^c^, p | 1.00 (ref) | 0.78 (0.52-1.18), 0.241 | 0.46 (0.27-0.78), 0.004 | 0.50 (0.33-0.77), 0.002 |

^a^ After excluding participants with any comorbidity as shown in Table 1.

^b^ per 100 person-years.

^c^ Adjustment with sex, age, education, marital status, income, residence, living with family, current smoking, current drinking, current exercise, regular intake of foods, comorbidities (except the excluded diseases themself), and ADL disability.

^d^ After excluding participants with hypertension or diabetes or heart diseases.
