# Supplementary material for: Association of changes in frailty status with the risk of all-cause mortality and cardiovascular death in older people: results from the Chinese Longitudinal Healthy Longevity Survey (CLHLS)
Source: BMC Geriatr. 2024 Jan 25;24:96. doi: 10.1186/s12877-024-04682-2 (PMC10809745; doi:10.1186/s12877-024-04682-2)
Supplement: Supplementary file 14 — Additional file 14: eTable 12. Association of changes in frailty status with cardiovascular death, after considering deaths of unknown reasons as cardiovascular death. [file 12877_2024_4682_MOESM14_ESM.docx]

eTable 12. Association of changes in frailty status with cardiovascular death, after considering deaths of unknown reasons as cardiovascular death

|  | Sustained pre/Frailty | Robustness to pre/Frailty | pre/Frailty to robustness | Sustained robustness |
| --- | --- | --- | --- | --- |
| No. of participants (n) | 812 | 493 | 432 | 1035 |
| Deaths (n) | 55 | 31 | 18 | 33 |
| Follow-up (PYs) | 2376.5 | 1714.6 | 1553.1 | 3866.7 |
| Mortality rate (95% CI)^a^ | 2.3 (1.7-2.9) | 1.8 (1.2-2.4) | 1.2 (0.6-1.7) | 0.9 (0.6-1.1) |
| Adjusted HR (95% CI)^b^, p | 1.00 (ref) | 0.62 (0.47-0.82), 0.001 | 0.47 (0.34-0.65), <0.001 | 0.42 (0.31-0.56), <0.001 |

^a^ per 100 person-years.

^b^ Adjustment with sex, age, education, marital status, income, residence, living with family, current smoking, current drinking, current exercise, regular intake of foods, comorbidities, and ADL disability.

Abbreviations: CI = confidence interval; HR = hazard ratio; PYs = person-years.
