# Supplementary material for: Association of changes in frailty status with the risk of all-cause mortality and cardiovascular death in older people: results from the Chinese Longitudinal Healthy Longevity Survey (CLHLS)
Source: BMC Geriatr. 2024 Jan 25;24:96. doi: 10.1186/s12877-024-04682-2 (PMC10809745; doi:10.1186/s12877-024-04682-2)
Supplement: Supplementary file 15 — Additional file 15: eTable 13. Association of changes in frailty status with cardiovascular death and all-cause mortality after multiple imputationa. [file 12877_2024_4682_MOESM15_ESM.docx]

eTable 13. Association of changes in frailty status with cardiovascular death and all-cause mortality after multiple imputation^a^

|  | Sustained pre/Frailty | Robustness to pre/Frailty | pre/Frailty to robustness | Sustained robustness |
| --- | --- | --- | --- | --- |
| *All-cause mortality* |  |  |  |  |
| No. of participants (n) | 971 | 580 | 517 | 1206 |
| Deaths (n) | 553 | 194 | 150 | 219 |
| Follow-up (PYs) | 2785.0 | 2009.0 | 1851.0 | 4464.0 |
| Mortality rate (95% CI)^b^ | 19.9 (18.4-21.3) | 9.7 (8.4-11.0) | 8.1 (6.9-9.4) | 4.9 (4.3-5.5) |
| Adjusted HR (95% CI)^c^, p | 1.00 (ref) | 0.62 (0.52-0.73), <0.001 | 0.54 (0.45-0.65), <0.001 | 0.42 (0.35-0.5), <0.001 |
|  |  |  |  |  |
| *Cardiovascular death* |  |  |  |  |
| No. of participants (n) | 971 | 580 | 517 | 1206 |
| Deaths (n) | 91 | 39 | 24 | 47 |
| Follow-up (PYs) | 2785.0 | 2009.0 | 1851.0 | 4464.0 |
| Mortality rate (95% CI)^b^ | 3.3 (2.6-3.9) | 1.9 (1.3-2.5) | 1.3 (0.8-1.8) | 1.1 (0.8-1.4) |
| Adjusted HR (95% CI)^c^, p | 1.00 (ref) | 0.69 (0.46-1.02), 0.061 | 0.49 (0.31-0.78), 0.003 | 0.47 (0.32-0.7), <0.001 |

^a^ Multiple imputation was performed by chained equations to create 10 datasets, of which the resultant model estimates for each were combined using Rubin`s rules.

^b^ per 100 person-years.

^c^ Adjustment with sex, age, education, marital status, income, residence, living with family, current smoking, current drinking, current exercise, regular intake of foods, comorbidities, and ADL disability.

Abbreviations: CI = confidence interval; HR = hazard ratio; PYs = person-years.
