# Supplementary material for: Association of changes in frailty status with the risk of all-cause mortality and cardiovascular death in older people: results from the Chinese Longitudinal Healthy Longevity Survey (CLHLS)
Source: BMC Geriatr. 2024 Jan 25;24:96. doi: 10.1186/s12877-024-04682-2 (PMC10809745; doi:10.1186/s12877-024-04682-2)
Supplement: Supplementary file 16 — Additional file 16: eTable 14. Association of changes in frailty status with cardiovascular death and all-cause mortality in participants with prefrailty or frailty at waves 2011 and 2014. [file 12877_2024_4682_MOESM16_ESM.docx]

eTable 14. Association of changes in frailty status with cardiovascular death and all-cause mortality in participants with prefrailty or frailty at waves 2011 and 2014

|  | Sustained frailty | Prefrailty to frailty | Frailty to prefrailty | Sustained prefrailty |
| --- | --- | --- | --- | --- |
| *All-cause mortality* |  |  |  |  |
| No. of participants (n) | 172 | 197 | 137 | 326 |
| Deaths (n) | 133 | 136 | 76 | 128 |
| Follow-up (PYs) | 386.0 | 518.3 | 407.6 | 1077.0 |
| Mortality rate (95% CI)^a^ | 34.5 (29.7-39.2) | 26.2 (22.5-30.0) | 18.6 (14.9-22.4) | 11.9 (10.0-13.8) |
| Adjusted HR (95% CI)^b^, p | 1.00 (ref) | 0.84 (0.65-1.08), 0.166 | 0.63 (0.47-0.84), 0.002 | 0.49 (0.37-0.65), <0.001 |
|  |  |  |  |  |
| *Cardiovascular death* |  |  |  |  |
| No. of participants (n) | 172 | 197 | 137 | 326 |
| Deaths (n) | 20 | 22 | 12 | 21 |
| Follow-up (PYs) | 386.0 | 518.3 | 407.6 | 1077.0 |
| Mortality rate (95% CI)^a^ | 5.2 (3.0-7.4) | 4.2 (2.5-6.0) | 2.9 (1.3-4.6) | 1.9 (1.1-2.8) |
| Adjusted HR (95% CI)^b^, p | 1.00 (ref) | 0.91 (0.47-1.75), 0.772 | 0.75 (0.36-1.59), 0.458 | 0.47 (0.23-0.95), 0.036 |

^a^ per 100 person-years.

^b^ Adjustment with sex, age, education, marital status, income, residence, living with family, current smoking, current drinking, current exercise, regular intake of foods, comorbidities, and ADL disability.

Abbreviations: CI = confidence interval; HR = hazard ratio; PYs = person-years.
