# Supplementary material for: Association of changes in frailty status with the risk of all-cause mortality and cardiovascular death in older people: results from the Chinese Longitudinal Healthy Longevity Survey (CLHLS)
Source: BMC Geriatr. 2024 Jan 25;24:96. doi: 10.1186/s12877-024-04682-2 (PMC10809745; doi:10.1186/s12877-024-04682-2)
Supplement: Supplementary file 17 — Additional file 17: eTable 15. Association of changes in frailty status with cardiovascular death and all-cause mortality in participants with robustness or frailty at waves 2011 and 2014. [file 12877_2024_4682_MOESM17_ESM.docx]

eTable 15. Association of changes in frailty status with cardiovascular death and all-cause mortality in participants with robustness or frailty at waves 2011 and 2014

|  | Sustained frailty | Robustness to frailty | Frailty to robustness | Sustained robustness |
| --- | --- | --- | --- | --- |
| *All-cause mortality* |  |  |  |  |
| No. of participants (n) | 172 | 121 | 83 | 1043 |
| Deaths (n) | 133 | 66 | 32 | 187 |
| Follow-up (PYs) | 386.0 | 354.0 | 285.1 | 3871.6 |
| Mortality rate (95% CI)^a^ | 34.5 (29.7-39.2) | 18.6 (14.6-22.7) | 11.2 (7.6-14.9) | 4.8 (4.2-5.5) |
| Adjusted HR (95% CI)^b^, p | 1.00 (ref) | 0.69 (0.50-0.95), 0.024 | 0.39 (0.26-0.58), <0.001 | 0.25 (0.18-0.33), <0.001 |
|  |  |  |  |  |
| *Cardiovascular death* |  |  |  |  |
| No. of participants (n) | 172 | 121 | 83 | 1043 |
| Deaths (n) | 20 | 15 | 5 | 41 |
| Follow-up (PYs) | 386.0 | 354.0 | 285.1 | 3871.6 |
| Mortality rate (95% CI)^a^ | 5.2 (3.0-7.4) | 4.2 (2.1-6.3) | 1.8 (0.2-3.3) | 1.1 (0.7-1.4) |
| Adjusted HR (95% CI)^b^, p | 1.00 (ref) | 1.01 (0.48-2.09), 0.984 | 0.44 (0.16-1.20), 0.109 | 0.39 (0.19-0.79), 0.009 |

^a^ per 100 person-years.

^b^ Adjustment with sex, age, education, marital status, income, residence, living with family, current smoking, current drinking, current exercise, regular intake of foods, comorbidities, and ADL disability.

Abbreviations: CI = confidence interval; HR = hazard ratio; PYs = person-years.
