# Supplementary material for: Association of changes in frailty status with the risk of all-cause mortality and cardiovascular death in older people: results from the Chinese Longitudinal Healthy Longevity Survey (CLHLS)
Source: BMC Geriatr. 2024 Jan 25;24:96. doi: 10.1186/s12877-024-04682-2 (PMC10809745; doi:10.1186/s12877-024-04682-2)
Supplement: Supplementary file 18 — Additional file 18: eTable 16. Association of changes in frailty status with cardiovascular death and all-cause mortality in participants with prefrailty or robustness at waves 2011 and 2014. [file 12877_2024_4682_MOESM18_ESM.docx]

eTable 16. Association of changes in frailty status with cardiovascular death and all-cause mortality in participants with prefrailty or robustness at waves 2011 and 2014

|  | Sustained prefrailty | Robustness to prefrailty | Prefrailty to robustness | Sustained robustness |
| --- | --- | --- | --- | --- |
| *All-cause mortality* |  |  |  |  |
| No. of participants (n) | 326 | 377 | 349 | 1043 |
| Deaths (n) | 128 | 103 | 91 | 187 |
| Follow-up (PYs) | 1077.0 | 1362.6 | 1268.0 | 3871.6 |
| Mortality rate (95% CI)^a^ | 11.9 (10.0-13.8) | 7.6 (6.2-9.0) | 7.2 (5.8-8.6) | 4.8 (4.2-5.5) |
| Adjusted HR (95% CI)^b^, p | 1.00 (ref) | 0.63 (0.48-0.82), 0.001 | 0.65 (0.49-0.85), 0.002 | 0.56 (0.44-0.71), <0.001 |
|  |  |  |  |  |
| *Cardiovascular death* |  |  |  |  |
| No. of participants (n) | 326 | 377 | 349 | 1043 |
| Deaths (n) | 21 | 21 | 13 | 41 |
| Follow-up (PYs) | 1077.0 | 1362.6 | 1268.0 | 3871.6 |
| Mortality rate (95% CI)^a^ | 1.9 (1.1-2.8) | 1.5 (0.9-2.2) | 1.0 (0.5-1.6) | 1.1 (0.7-1.4) |
| Adjusted HR (95% CI)^b^, p | 1.00 (ref) | 0.77 (0.41-1.43), 0.408 | 0.53 (0.26-1.09), 0.084 | 0.70 (0.40-1.24), 0.220 |

^a^ per 100 person-years.

^b^ Adjustment with sex, age, education, marital status, income, residence, living with family, current smoking, current drinking, current exercise, regular intake of foods, comorbidities, and ADL disability.

Abbreviations: CI = confidence interval; HR = hazard ratio; PYs = person-years.
